# NMR characterisation of the minimal interacting regions of centrosomal proteins 4.1R and NuMA1: effect of phosphorylation

**DOI:** 10.1186/1471-2091-11-7

**Published:** 2010-01-28

**Authors:** Miguel A Treviño, Mar Rodríguez-Rodríguez, Isabel Correas, Miguel Marcilla, Juan P Albar, Manuel Rico, M Ángeles Jiménez, Marta Bruix

**Affiliations:** 1 Departamento de Espectroscopía y Estructura Molecular, Instituto de Química Física Rocasolano, Consejo Superior de Investigaciones Científicas, Serrano 119, 28006 Madrid, Spain; 2 Departamento de Biología Molecular, Centro de Biología Molecular Severo Ochoa, Consejo Superior de Investigaciones Científicas y Universidad Autónoma de Madrid, Nicolás Cabrera 1, Cantoblanco, 28049 Madrid, Spain; 3 Departamento de Proteómica, Centro Nacional de Biotecnología, Consejo Superior de Investigaciones Científicas, Darwin 3, Cantoblanco, 28049 Madrid, Spain

## Abstract

**Background:**

Some functions of 4.1R in non-erythroid cells are directly related with its distinct sub-cellular localisation during cell cycle phases. During mitosis, 4.1R is implicated in cell cycle progression and spindle pole formation, and co-localizes with NuMA1. However, during interphase 4.1R is located in the nucleus and only partially co-localizes with NuMA1.

**Results:**

We have characterized by NMR the structural features of the C-terminal domain of 4.1R and those of the minimal region (the last 64 residues) involved in the interaction with NuMA1. This subdomain behaves as an intrinsically unfolded protein containing a central region with helical tendency. The specific residues implicated in the interaction with NuMA1 have been mapped by NMR titrations and involve the N-terminal and central helical regions. The segment of NuMA1 that interacts with 4.1R is phosphorylated during mitosis. Interestingly, NMR data indicates that the phosphorylation of NuMA1 interacting peptide provokes a change in the interaction mechanism. In this case, the recognition occurs through the central helical region as well as through the C-terminal region of the subdomain meanwhile the N-terminal region do not interact.

**Conclusions:**

These changes in the interaction derived from the phosphorylation state of NuMA1 suggest that phosphorylation can act as subtle mechanism of temporal and spatial regulation of the complex 4.1R-NuMA1 and therefore of the processes where both proteins play a role.

## Background

The protein 4.1R was originally described as a component of red blood cells essential in the maintenance of cellular shape and integrity. In these cells, protein 4.1R is an 80 kDa component that anchors the spectrin-actin network to the overlaying lipid bilayer through interactions with cytoplasmic domains of transmembrane proteins [1,2]. In non-erythroid cells the expression pattern of protein 4.1R is more complex and multiple isoforms of 4.1R are mainly produced as a result of extensive alternative splicing of the 4.1R pre-mRNA [3-7]. Non-erythroid 4.1R has been found adjacent to the cellular membrane but also in different intracellular regions such as the nucleus or the centrosome indicating that 4.1R possesses a wider range of functions beyond that of maintaining the cell shape [2,8]. It has been described that 4.1R is essential for microtubule dynamics, maintenance of centrosome integrity, cell cycle progression and correct formation of mitotic spindles among others [9]. 4.1R is an adaptor protein and these functions may be related with its capacity to interact with different partners. In this sense, it could serve to integrate centrosomal components and thus be critical for some centrosomal functions, such as regulation of polarity, intracellular transport, etc. Protein 4.1R interacts with NuMA1 [10] and 4.1R depletion provokes mislocalization of NuMA1 [9]. The changes in the expression profile of both proteins alter the cell cycle and perturb cell mitotic spindles in the same way [9-12]. This fact suggests that the 4.1R and NuMA1 functions related to cell cycle progression and spindle pole integrity might be a consequence of their interaction.

**Figure 1:**
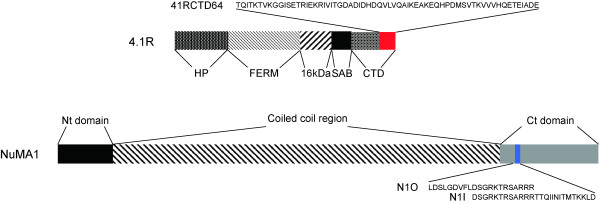
**Schematic representation of the different domains of 4.1R and NuMA1**. Coloured segments correspond to the minimal interacting regions and the amino acid sequences of the 4.1R subdomain and NuMA1 peptides used in this study are shown. Nomenclature of 4.1R domains: HP: Head Piece; FERM: 4.1, Ezrin, Radixin, Moesin; 16 kDa: 16 kDa; SAB: Spectrin/Actin Binding domain; CTD: C-terminal domain.

Prototypical non-erythroid protein 4.1R is constituted by 5 domains (Figure 1), 1) Head Piece (HP); 2) 4.1, Ezrin, Radixin, Moesin (FERM); 3) 16 kDa; 4) Spectrin/Actin Binding domain (SAB); and 5) C-terminal domain (CTD) [13,14]. In turn, NuMA1 has three domains (Figure 1), two terminal domains (1-207 and 1729-2115) and a central long coiled-coil region [15]. The interaction between 4.1R and NuMA1 occurs between the C-terminal domains of both proteins. The interacting regions were mapped by yeast two hybrid assay and the minimal interacting epitopes were defined as the last 64 amino acids for 4.1R and the region comprised by the amino acids in positions 1788 to 1808 for NuMA1 [10] (Figure 1). *In vivo*, during mitosis, 4.1R and NuMA1 co-localize in the centrosome where both proteins have been found to be phosphorylated [16-18] but in other cell cycle steps, e.g. in interphase, 4.1R immunoreactivity is diffused in the nucleus and cytoplasm and concentrated in nuclear speckles enriched in splicing factors [19]. Nuclear protein 4.1R only partially co-localizes with nuclear NuMA1 [10]. Whether or not these two proteins interact in the nucleus or in the centrosome, remains to be established. Similarly, there are a number of open questions: i) is a control of the interaction of 4.1R and NuMA necessary for their centrosomal location and therefore for the cell cycle progression?, ii) is the fine interaction regulated at each moment of the cell cycle?, iii) how does phosphorylation affect the interaction of both proteins? Here, our goals are to characterize at high resolution the minimal region within the 4.1R C-terminal domain involved in the interaction with NuMA1 and to determine whether phosphorylation in the interacting regions regulates the 4.1R-NuMA1 interaction.

## Results

### CD and NMR study of the C-terminal domain of 4.1R

**Figure 2:**
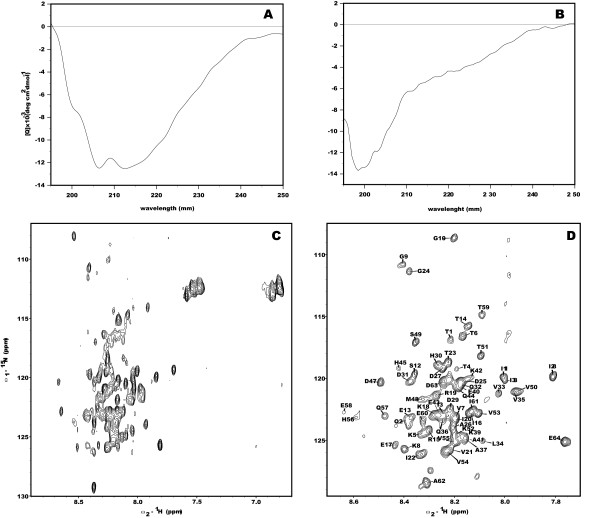
**CD and ^1^H-^15^N HSQC spectra of 4.1R domains**. A: CD spectra of the C-terminal domain of 4.1R. B: CD spectra of the 4.1R-CTD64 domain of 4.1R. Samples for CD were 5 mM in 10 mM Na_2_HPO_4 _at pH 6.5 (4.1R C-terminal domain) or pH 4.9 (4.1R-CTD64). C: ^1^H-^15^N HSQC spectra of the C-terminal domain of 4.1R at pH 6.5 (0.6 mM protein in H_2_O 90%/D_2_O 10% at pH 6.5). D: ^1^H-^15^N HSQC spectra of the 4.1R-CTD64 domain at pH 4.9 (0.1 mM protein in Na_2_HPO_4 _100 mM in 90% H_2_O/10% D_2_O pH4.9) with signal assignments. Note that the spectral width in D is smaller than in C to facilitate the visualization of the assignments.

The complete C-terminal domain of 4.1R expressed well and was soluble at pH 6.5. The CD spectrum of the domain (Figure 2A) indicates that the protein has a high level of secondary structure (12% α-helix; 43% β-strand and 45% random coil). This result agrees with the proportions obtained from the secondary structure predictor Jpred [20] (11% α-helix; 32% β-strand; 57% random coil). However, the ^15^N-HSQC spectrum (Figure 2C), shows a lack of signal dispersion typical of unstructured protein sequences. Besides this poor dispersion, the widths of the NMR signals are not uniform, some of them are very broad and others are very sharp. In addition, the number of signals in the spectrum is less than what is expected for this domain indicating that different dynamic regimes due to chemical or conformational exchange are present. All these data suggest the presence of possible aggregation states that makes impossible the NMR assignment of the complete C-terminal domain of 4.1R. Therefore, we focussed on a shorter form of the C-terminal domain of 4.1R.

### NMR Structural studies of the subdomain 4.1R-CTD64 and of NuMA1 peptides

A shorter form of the C-terminal domain of 4.1R containing the last 64 amino acids (4.1R-CTD64), which has been reported to be the minimal unit capable of interacting with NuMA1 [10], was produced.

**Figure 3:**
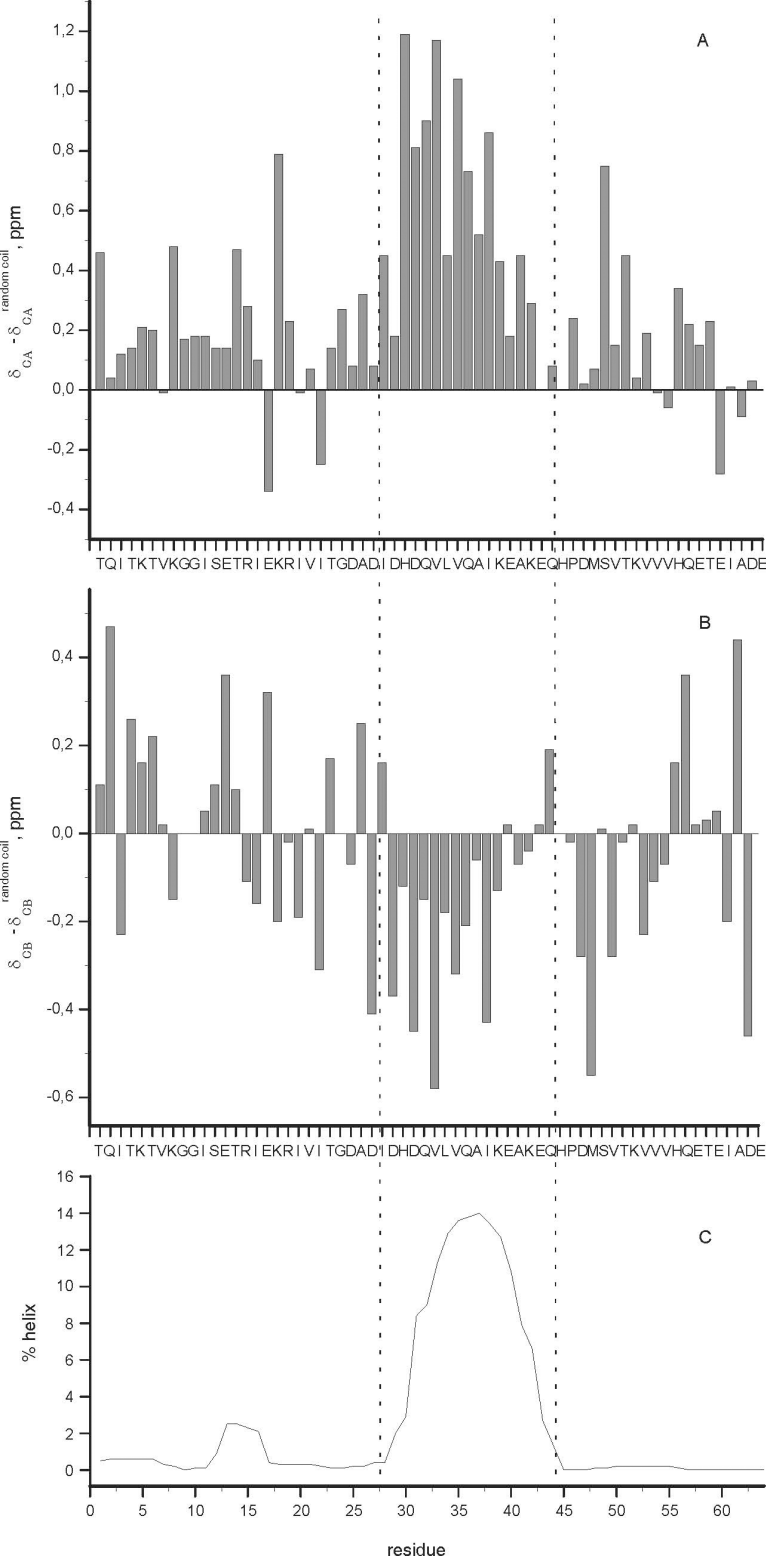
**Tendency of 4.1R-CTD64 to adopt an α-helix conformation**. A: Conformational Δδ^13^C_α _(Δδ^13^C_α _= δ^13^C_α, experimental_- δ^13^C_α, random coil_, ppm) chemical shifts as a function of sequence. Positive values indicate α-helix structure. B: Conformational Δδ^13^C_β _(Δδ^13^C_β _= δ^13^C_β, experimental_- δ^13^C_β, random coil_, ppm) chemical shifts as a function of sequence. Negative values indicate α-helix structure. C: Theoretical prediction of helical percentage at 5°C and pH 5.0 by AGADIR [23] as a function of residue number.

The ^15^N-HSQC spectrum of 4.1R-CTD64 shows poor signal dispersion, suggesting the absence of preferred structure in agreement with the CD data (Figure 2B). In contrast to the full length C-terminal domain, the majority of the 4.1R-CTD64 NMR signals have similar widths and the number of cross peaks matches that expected on the basis of the 4.1R-CTD64 sequence. In these conditions, the complete assignment of its backbone was carried out (Figure 2D). The analysis of the conformational chemical shifts (Δδ^13^C = δ^13^C_, experimental_- δ^13^C_, random coil_, ppm) [21], shows consistent positive or negative values, for C_α _and C_β _respectively, between residues 28 and 44 compatible with the presence of an α-helix conformation in this region (Figure 3A and 3B). The mean helix percentage for the segment 28-44, calculated on the bases of the Δδ^13^C_α _values is 15% [22]. This experimental data is in the range of that predicted by AGADIR [23], a mean helix population of 9% in this region (Figure 3C).

**Figure 4:**
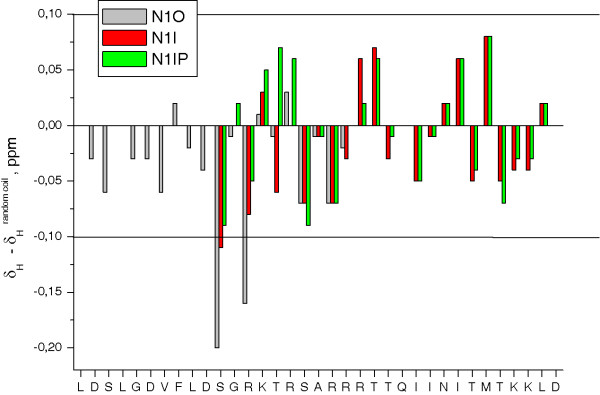
**Conformational shifts for the Hα protons of NuMA1 peptides**. Histogram showing the conformational shifts for the Hα protons (Δδ = δ_experimental _- δ_random coil_, ppm) of control peptide (N1O, grey bars), non-phosphorylated NuMA peptide (N1I, red bars) and phosphorylated NuMA peptide (N1IP, green bars) as a function of peptide sequence. The Δδ range indicative of random coil peptides is shown by lines at 0.1 and -0.1 ppm. N1O: sequence 1776-1796 of NuMA1, N1I: sequence 1785-1810 of NuMA1, N1IP: same as N1I but phosphorylated in T6 corresponding to T1790 of NuMA1.

**Figure 5:**
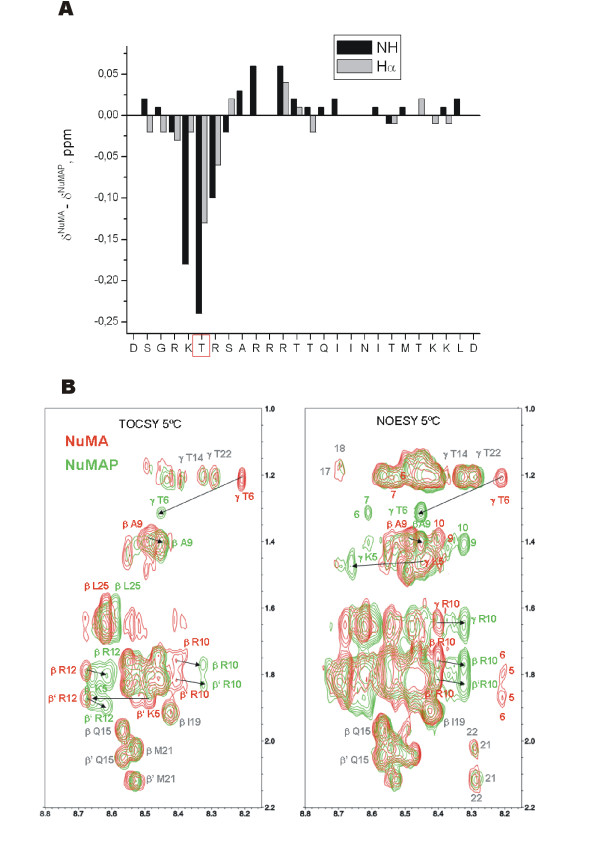
**NMR results for N1I and N1IP**. A) Histogram showing the chemical shift differences between non-phosphorylated (N1I) and phosphorylated NuMA1 peptide (N1IP) for the NH (black bars) and Hα (grey bars) protons as a function of peptide sequence. B) TOCSY and NOESY spectral regions of non-phosphorylated (red contours) and phosphorylated (green contours) NuMA1 peptides. Signals that change upon phosphorylation are indicated in both spectra. Signal labels are red for the non-phosphorylated peptide, green for the phosphorylated peptide and grey for those signals that does not change with phosphorylation. Intrarresidual signals are labelled with the proton involved in the interaction with the corresponding amide HN (greek symbol), the one letter amino acid code and the residue number. In the NOESY spectra, interresidual signals are labelled with two numbers corresponding to the aliphatic proton of residue *i *and the HN proton of residue *i+1*. N1I: sequence 1785-1810 of NuMA1, N1IP: same as N1I but phosphorylated in T6 corresponding to T1790 of NuMA1.

The ^1^H NMR spectra (COSY, TOCSY and NOESY) of all NuMA1 peptides (NuMA1 overlapping, N1O; NuMA1 Interacting, N1I and phosphorylated NuMA1 Interacting, N1IP) (pH 4, 5°C), were analysed and assigned. The deviation of the ^1^H conformational chemical shifts [21] of the three species are all < 0.1 ppm, except for those shown by two residues in the control peptide (N1O) and one in the non-phosphorylated peptide (N1I) (Figure 4). This indicates that the peptides do not have any significant tendency to adopt secondary structure. Figure 5A shows the differences in the chemical shifts of HN and Hα protons between the phosphorylated (N1IP) and non-phosphorylated peptide (N1I). As expected, the largest chemical shift differences are observed for T6 (the phosphorylated amino acid) and adjacent residues, but significant changes are also found for R10 and R12. All other NMR signals show a remarkably good superposition, as seen in the TOCSY and NOESY spectral region shown in Figure 5B.

**Figure 6:**
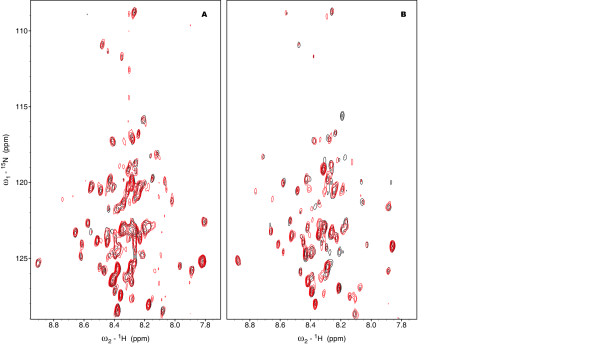
**Titration of 4.1RCTD64 with N1O peptide at pH 4.9 and with N1I at pH 4.0**. Titration of ^15^N labelled 4.1R-CTD64 followed by ^1^H-^15^N HSQC NMR spectra at 25°C. A: Titration with N1O (sequence 1776-1796 of NuMA1) at pH 4.9. B: Titration with N1I (sequence 1785-1810 of NuMA1) at pH 4.0. In both cases black lines belong to the NMR spectra of 4.1R-CTD64 alone and red lines to the spectra in presence of NuMA1 peptides (ratio 1:20).

4.1R-CTD64 has a theoretical pI of 5.2 meanwhile the NuMA1 interacting peptide (N1I) has a pI of 11.8. To assess if the difference of charge is leading to their interaction via electrostatic forces, we first titrated 4.1R-CTD64 with NuMA1 overlapping (N1O) peptide, which overlaps with N1I and has a similar pI (11.4). We added up to 20:1 N1O to 4.1R-CTD64, in the same conditions used for N1I (see below), without changes in the ^15^N-HSQC spectrum of the domain (Figure 6A). In all cases titrations were followed by chemical shift perturbation analysis [24,25].

First attempts to titrate 4.1R-CTD64 with N1I were made at pH 4, where both 4.1R-CTD64 and N1I are completely soluble. In these conditions, even at an excess of N1I:4.1R-CTD64, 20:1, just slight changes in the NMR spectra were detected, indicating absence of interaction between both species in these conditions (Figure 6B).

**Figure 7:**
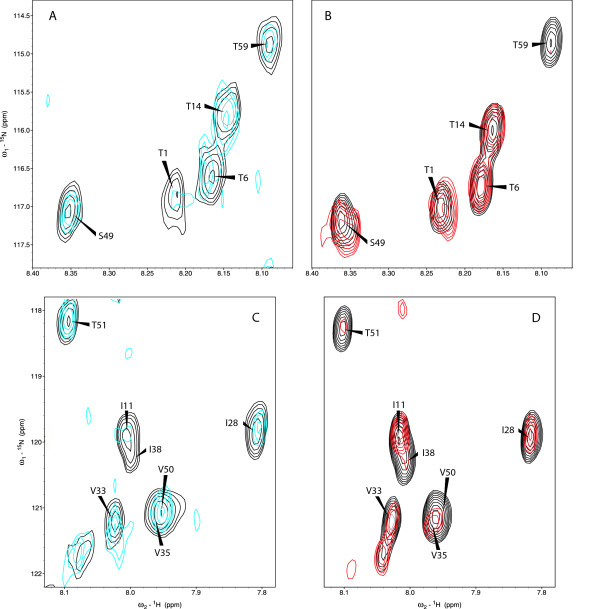
**Detail of the ^1^H-^15^N HSQC spectra corresponding to the titrations of 4.1RCTD64 with NuMA1 peptides at pH 4.9**. A and B in one hand and C and D in the other show the same regions of the spectra. A and C: Spectra of 4.1R-CTD64 alone (black) or in presence of 20 to 1 ratio of N1I (sequence 1785-1810 of NuMA1) (blue). B and D: Spectra of 4.1RCTD64 alone (black) or in presence of 20 to 1 ratio of N1IP (N1I phosphorylated) (red). Note the drastic changes in the signal intensity of T51 and T59 after titration with N1IP, of I11 after titration with N1I, or of I38 after titration with both peptides.

**Figure 8:**
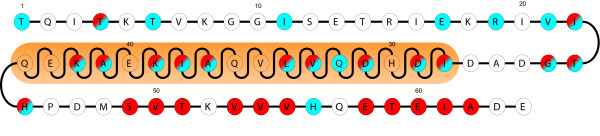
**Representation of the interaction of 4.1R-CTD64 with NI1 and NI1P peptides from NuMA1**. Graphic representation of the interaction of 4.1R-CTD64 with NI1 (sequence 1785-1810 of NuMA1) and NI1P (N1I phosphorylated) peptides from NuMA1. Residues coloured in blue indicate changes after titration with N1I. Residues coloured in red indicate changes after titration with N1IP. Residues in both colours indicate changes after both titrations. The region with α-helix propensity as seen by NMR chemical shift analysis is represented with undulating lines within an orange cylinder.

Taking into account that at pH 4 Asp and Glu residues are partially titrated, and that the electrostatic forces can affect their implication in the interaction, titrations were repeated at pH 4.9, where the acid residues are almost completely in their carboxylate state and 4.1R-CTD64 is still soluble enough to yield a fair spectrum. In these conditions, changes in the ^15^N-HSQC spectrum of 4.1R-CTD64 were detectable even at 5:1 of N1I:4.1R-CTD64 (Figure 7). The residues of 4.1R-CTD64 participating in the interaction (T1, T4, T6, I11, E17, R19, V21, I22, T23, G24, I28, D29, D31, Q32, V33, L34, A37, I38, K39, A41, K42, H45, H56) are mainly concentrated in the N-terminal and central portion of the domain except for H56, and no effect was detected for the last 19 amino acids (Figure 8).

Proteomic studies show that NuMA1 is phosphorylated during mitosis at position 1790 [16], which corresponds to T6 in the sequence of the N1I peptide. To evaluate the effect of the phosphorylation of NuMA1 in the interaction, 4.1R-CTD64 at pH 4.9 was titrated with phosphorylated NuMA1 peptide (N1IP). The changes observed in the NMR spectra indicate the interaction of the peptide with many residues of 4.1R-CTD64 (T4, I22, T23, G24, D29, D31, V33, L34, V35, A37, I38, K39, A41, K42, H45, S49, V50, T51, V53, E58, T59, E60, I61, A62). Many of them, as in the case of the titration with the non-phosphorylated N1I, are in the central region of the domain but alterations in the position of nuclei belonging to some C-terminal residues were also detected. The disappearance of specific signals in the ^15^N-^1^H HSQC spectrum takes place at similar peptide to 4.1R-CTD64 ratios in both cases.

In short, the titrations with both peptides provoke changes in 15 common amino acids, mainly sited in the central region of 4.1R-CTD64. Interestingly, the non common affected residues are located in the opposite ends of the domain. Thus, the interaction with N1I, exclusively affects 8 additional residues in the N-terminal portion of 4.1R-CTD64 whereas the titration with N1IP, perturbs other 11 residues which are exclusively located in the C-terminal region of 4.1R-CTD64.

## Discussion

The NMR chemical shift data of 4.1R C-terminal domain presented here indicates that the domain is intrinsically unstructured. Intrinsically unstructured proteins (IUP) and domains have gained prominence in recent years due to their importance in regulation, signalling, and other processes where a subtle and precise control is necessary [26]. In this regard, 4.1R and NuMA1 are involved in cell cycle control and mitosis, two processes where fine temporal and spatial coordination is needed and where a high number of natively unstructured proteins has been identified [26]. The lack of structure provides functional advantages as binding promiscuity [27], moonlighting [28], decoupled specificity and affinity [29], the possibility to be protein interaction hubs [30], accessibility to post-translational modifications [31] or a higher capture radius and high speed of the interaction even at low concentrations [32] that can be essential for the functions of the 4.1R C-terminal domain in cell cycle and mitosis.

This lack of preferred three dimensional structure, as seen by NMR, is compatible with the CD data showing that the C-terminal domain of 4.1R conserves elements of secondary structure. This is not an uncommon situation for unstructured proteins were the grade of disorder can be very variable and many states, from conformational ensembles of completely unstructured proteins to mostly folded proteins with disorder only in small regions have been described. Between these two extremes all intermediate situations can be found [33]. Additionally, the absence of some expected signals and the different signal widths as observed in the ^15^N-HSQC spectrum of the C-terminal domain of 4.1R can be attributed to aggregation processes. Predictions of aggregation made by TANGO [34] indicate short regions with low tendency for β-aggregation and no detectable α-aggregation. These processes could increase at the mM concentrations used in NMR spectroscopy. From a solution NMR perspective, a protein or protein domain associated forming large entities tumbles as part of a large complex, which results in signal broadening and poor sensitivity. In the same way, the different regions of an unstructured protein being implicated in aggregation have broad peaks that can be unobservable due to the large molecular weight dynamic regime and/or possible intermediate exchange regime.

In any case, these problems preclude the assignment of the complete C-terminal domain of 4.1R, essential for subsequent studies of interaction by NMR. As a consequence, we centred our study on a shorter form containing only the last 64 amino acids, 4.1R-CTD64, which has been defined as the minimal subdomain able to interact with NuMA1 [10]. The NMR, as well as the CD data, indicate that the 4.1R-CTD64 subdomain is also unstructured but, based on the Δδ^13^Cα, there is a region (from 28 to 44) with tendency to form an α-helix in aqueous solution.

By using the chemical shift mapping methodology [24,25], we have determined that 4.1R-CTD64 interacts with NuMA1 peptide (N1I) at pH 4.9 through the N-terminal residues as well as the central region. Interestingly, this central region coincides with the segment which has tendency to be α-helical. The NMR assignment of 4.1R-CTD64 at pH 3 and pH 4.9 shows that the tendency to form α-helix in the region 28 to 44 is almost the same in both conditions, indicating that the observed α-helix population does not depend on the protonation state of the carboxylate groups. However, the interaction between 4.1R-CTD64 and N1I is strongly affected by the charges present in the peptide, as no interaction was detected during the titration of 4.1R-CTD64 with N1I at pH 4 but changes are important at pH 4.9.

Regarding the nature of the forces driving the interaction, the electrostatic charges seem not to be determinant because the overlapping peptide N1O, which shares the N-terminal sequence with N1I and has a similar pI, does not interact with 4.1R-CTD64. This also suggests that the C-terminal region of N1I is key for the interaction. This region of N1I contains a high number of hydrophobic residues which have been reported to be preferentially used in interactions by unstructured proteins [35].

Proteomic studies show that NuMA1 is phosphorylated at position 6 of N1I during mitosis (position 1790 in complete NuMA1) [16]. During this phase of the cell cycle, NuMA1 and 4.1R co-localize in the centrosome while 4.1R only partially co-localizes with NuMA1 at interphase [10]. Our data show that the interaction of 4.1R-CTD64 with the N1I peptide differs depending on the phosphorylation state. The same residues of the central region of 4.1R-CTD64 interact with both peptides, but there are differences in the participation of both termini: residues of the N-terminal portion of 4.1R-CTD64 interact with the non-phosphorylated N1I while residues from the C-terminal region are affected during the titration with the phosphorylated N1IP. In both cases, no differences in the regime of interaction are detected regarding to its affinity.

Two principal kinds of non-mutually exclusive elements of interaction have been postulated in IUPs: molecular recognition features and preformed elements. Molecular recognition features are short regions that undergo a disorder to order transition that is stabilized by binding to their partners [36]. On the other hand, preformed elements are elements of secondary structure which are present in the free IUP form that usually are the first interacting element, and that maintain their structure after interaction [37]. In our case, the 28-44 region shares characteristics of both kinds of recognition elements as a preformed structure, the α-helix, is present though not 100% populated. The fact that the same residues in this central region are affected in both titrations suggests that the phosphorylated residue of NuMA1 is not directly contacting with the α-helix but more probably with the C-terminal portion of 4.1R-CTD64, where the residues exclusively affected during the titration with N1IP are located.

The identification of the elements responsible for the interactions in unstructured proteins is of major importance because even subtle differences are able to produce key changes. It has been described that the initial steps of these interactions are driven by just a few number of residues, even with a low affinity, and then the remaining regions start to contact with the partner [32,36,37]. In this context it is not difficult to think that even minor differences in the recognition are important enough to drive a completely different interacting surface. Related to this, a possibility is that after the initial contact by an element of interaction, the unstructured protein can adopt different structural dispositions that can produce different effects such as activation or inhibition of the partner [28]. Clearly, this can be the case for the interaction of 4.1R-CTD64 and N1I and N1IP.

From our data we cannot deduce if 4.1R and NuMA1, which co-localize at the centrosome and partially co-localize in the nucleus, are interacting at both intracellular sites, nucleus and centrosome, or only at the centrosome. A scenario compatible with our data is that both proteins bind each other in the nucleus leaving the phosphorylatable threonine accessible to protein kinases. NuMA1 could be phosphorylated previously to mitosis, which can trigger the reorientation or reorganization of both proteins forming the complex. In this new state they could participate in spindle pole formation. The two different ways of interaction may also be related with different functions, in a process of moonlighting consequence of the state of phosphorylation. A similar kind of process has been described in IUPs [28], like for example for the IUP Cystic Fibrosis Transmembrane-conductance Regulator (CFTR) which interacts with the chloride channel and activates or inhibits it as a function of its phosphorylation state [38].

## Conclusions

In conclusion, the C-terminal domain of 4.1R, as well as the subdomain 4.1R-CTD64, have all features of intrinsically unstructured domains. By using the NMR data presented here, we were able to identify the residues involved in the interaction with N1I of NuMA1. Even more, we have found that 4.1R-CTD64 binds the phosphorylated form of N1I (N1IP) in a different way. Taking into account that the phosphorylated form of NuMA1 is detected in mitosis, where both proteins may interact, we can suggest that the changes produced by phosphorylation modulate the interaction and therefore the progression of cell cycle and the spindle pole formation. Other phosphorylations, even in regions different from this one [17] can aid the more precise modulation of its interaction. In any case, the results presented here open the door to study the interaction between the complete proteins or functional domains regarding the influence of the phosphorylation state, and how the reorientation of both molecules as a consequence of the phosphorylation affects their function in mitosis.

## Methods

### Protein production and purification

A plasmid containing the complete 4.1R sequence [39] was used as template in PCRs specific for 4.1R C-terminal domain (last 174 amino acids of human 4.1R) and 4.1R-CTD64 (last 64 amino acids): (TQITKTVKGGISETRIEKRIVITGDADIDHDQVLVQAIKEAKEQHPDMSVTKVVVHQETEIADE). PCRs (30 cycles 1 min 95°C; 1 min 52°C; 1 min 72°C) were carried out with the Hot start KOD polymerase (Merck, Germany) according manufacturer recommendations using 5'-GCGGCGATGAGTGTCTCTGCATGGAGTCTGTACCAGAA-3' (for the complete C-terminal domain) or 5'-GCGCATATGACTCAAATTACCAAGACTGT-3' (for 4.1R-CTD64) and 5'-GGGCTCGAGTCATTACTCATCAGCAATCTCGGT-3' (for both fragments) as primers. PCR products were sequentially digested for 3 hours at 60°C with BtgZI and then with BamHI at 37°C (complete domain) or simultaneously at 37°C with NdeI and BamHI (64 amino acids subdomain). The digested fragments were cloned in a modified pET15b vector with the thrombin cleavage site replaced with a TEV protease cleavage site.

(His)_6_-tagged proteins were expressed in *E. coli *strain BL21 (DE3). Production was made by High Cell Density Fermentation [40] in 700 ml of HCDF medium in a Bioflo 110 fermentor (New Brunswick Scientific, NJ). Labelled proteins were obtained using HCDF medium containing ^15^NH_4_Cl and ^13^C-u-glucose as exclusive nitrogen and carbon sources, respectively. Collected bacteria were resuspended in 40 ml of NaH_2_PO_4 _50 mM pH 7, NaCl 200 mM containing complete protease inhibitor without EDTA (Roche, Germany) and lysed by sonication. Lysates were purified by nickel affinity chromatography and fractions were eluted with lysis buffer containing 300 mM imidazole. Eluates were dialyzed against 50 mM NaH_2_PO_4 _pH 7, 200 mM NaCl and 10 mM PMSF and digested with TEV protease overnight at 4°C. The proteins were subjected to a second purification step by nickel affinity chromatography and the overexpressed proteins lacking (His)_6_-tag were collected from the throughput and re-dialyzed against appropriate buffers for the subsequent experiments.

### Peptides

Synthetic peptides NuMA1 interacting (N1I, comprising residues 1785-1810 of NuMA1), Ac-DSGRKTRSARRRTTQIINITMTKKLD-NH_2_, and phosphorylated NuMA1 interacting (N1IP) peptides, Ac-DSGRKT(p)RSARRRTTQIINITMTKKLD-NH_2 _were obtained from Caslo (Denmark). The NuMA1 overlapping peptide (N1O, encompassing residues 1776-1796 of NuMA1) Ac-LDSLGDVFLDSGRKTRSARRR-NH_2 _was synthesized on an automated multiple peptide synthesizer (Multipep, Intavis AG, Koln, Germany) using the solid-phase procedure and standard Fmoc chemistry [41]. Side chain protecting groups were as follows: OtBu (D), tBu (S, T), Pbf (R) and Boc (K). After synthesis, protecting side chain groups were removed, and the peptide was cleaved from the resin following the method of King et al. [42]. Peptides were purified by high-performance liquid chromatography (HPLC) on a DeltapaK C18 (7,8 × 300 mm) semi-preparative column (Waters). Peptide homogeneity and integrity were confirmed by analytical HPLC, amino acid analysis (Beckman 6300 amino acid analyzer, after acid hydrolysis in a N_2 _atmosphere for 18 h at 110°C), mass spectrometry (4800 Applied Biosystems, MALDI-TOF-TOF, matrix-assisted laser desorption-ionization time-of-flight mass spectrometer) and by NMR analysis.

### CD spectra

CD spectra were obtained in a JASCO J-810 spectropolarimeter equipped with a Peltier type cell holder. Protein concentration were in all cases 5 μM (1-mm pathlength; far-UV region). Each spectrum (0.2-nm intervals) was the average of 3-4 measurements with different samples (each of them the average of 4 scans) performed at a rate of 20 nm min^-1 ^using a response time of 2 s and a bandwidth of 1 nm. The temperature was kept constant at 20°C. The buffer contribution to the CD spectrum was subtracted from the experimental data, and the corrected ellipticities were converted to mean residue ellipticities. Samples contained 10 mM Na_2_HPO_4 _at pH 6.5 (4.1R C-terminal domain) or pH 4.9 (4.1RCTD64).

### NMR spectra acquisition and analysis

Samples for NMR were prepared at 0.6 mM in H_2_O 90%/D_2_O 10% at pH 6.2 (4.1R C-terminal domain), at 0.1 mM in Na_2_HPO_4 _100 mM in 90% H_2_O/10% D_2_O pH 3.0, 4.0 or 4.9 (4.1R-CTD64) or at 1 mM in 90% H_2_O/10% D_2_O pH 4.0 for N1I, N1IP, and NIO. For the titration, peptides were prepared in the same buffer as the 4.1R-CTD64 sample. All samples contained sodium 4,4-dimethyl-4-silapentane-1-sulfonate (DSS) as the chemical shift reference. Spectra were acquired on a Bruker Avance 800 MHz spectrometer equipped with a z-gradient cryoprobe. 3D HNCA, HN(CO)CA, CBCANH and CBCA(CO)NH spectra were acquired for the assignment of the backbone of 4.1R-CTD64 at pH 3.0. The standard assignment methodology was followed [43]. All assignments were transferred to the pH 4.9 conditions and the correct assignment was confirmed using a set of β- and γ-carbon edited ^1^H-^15^N spectra. These experiments classify peaks according to the number of β and γ hydrogens. Compared to a standard HSQC spectra, these NMR experiments yield a series of HSQC sub-spectra with greatly reduced overlap as peaks are classified as a function of the type of amino acid as well as the type of the preceding one [44].

For NuMA1 peptides, 2D COSY, TOCSY and NOESY spectra were acquired and assigned following standard methods [45].

## Authors' contributions

MAT carried out the molecular biology and biophysical experiments, produced the proteins and participated in the design of the study. MRR participated in the molecular biology procedures. IC provided the plasmid containing 4.1R. MM synthesized the peptides. JPA coordinated the peptide synthesis. MR conceived the study and participated in its coordination. MAJ assigned the peptides spectra, and participated in the coordination of the study. MB participated in the assignment of 4.1RCTD64, conceived the study, participated in its design and coordination. All authors helped to draft and discussed and approved the final manuscript.
